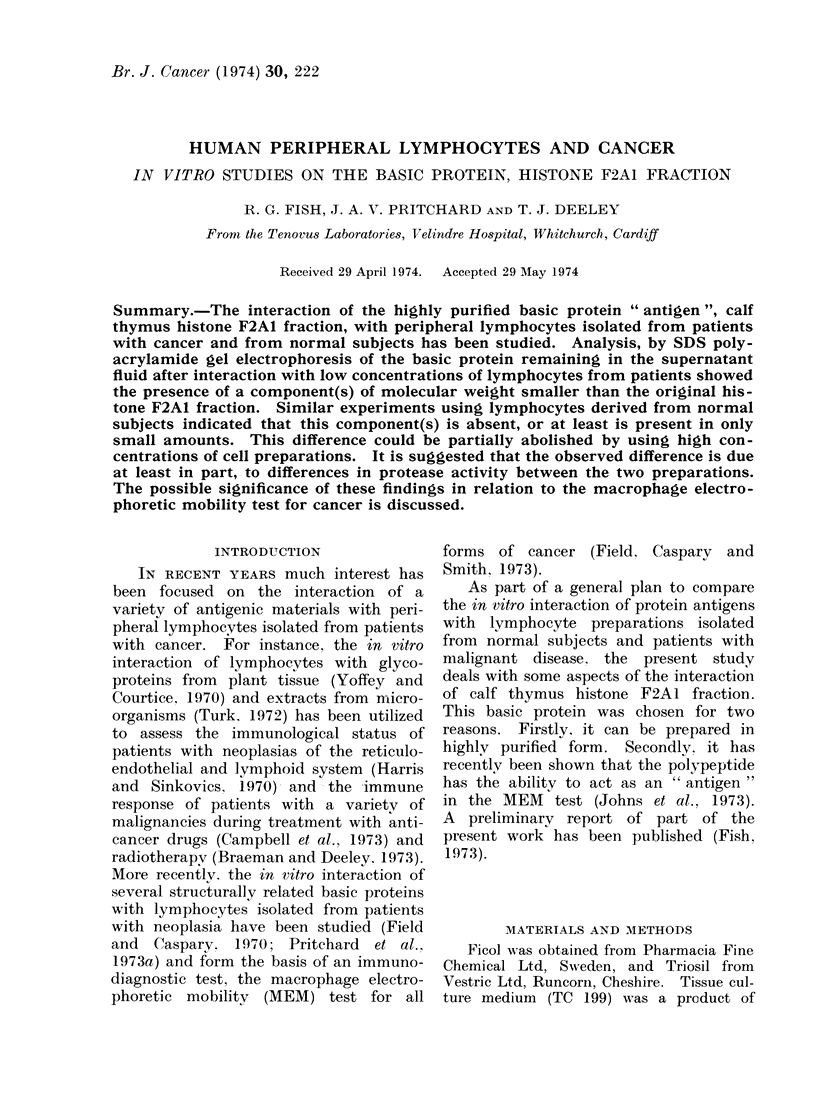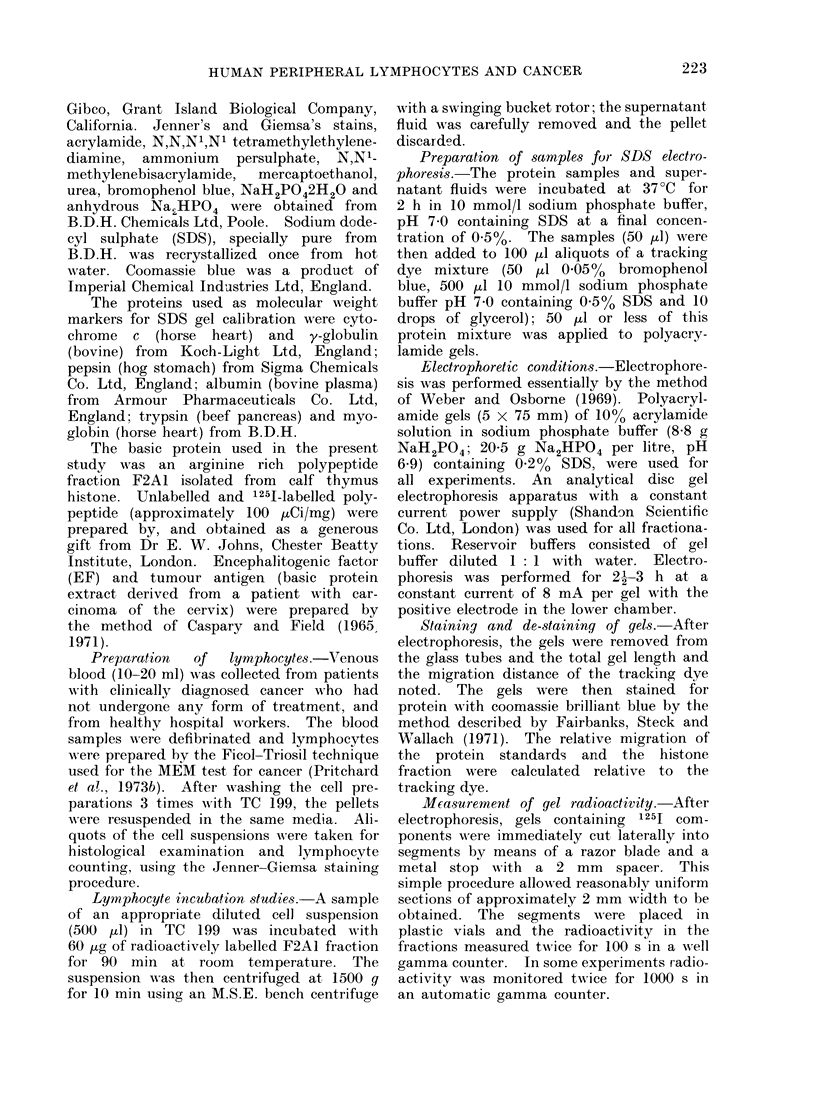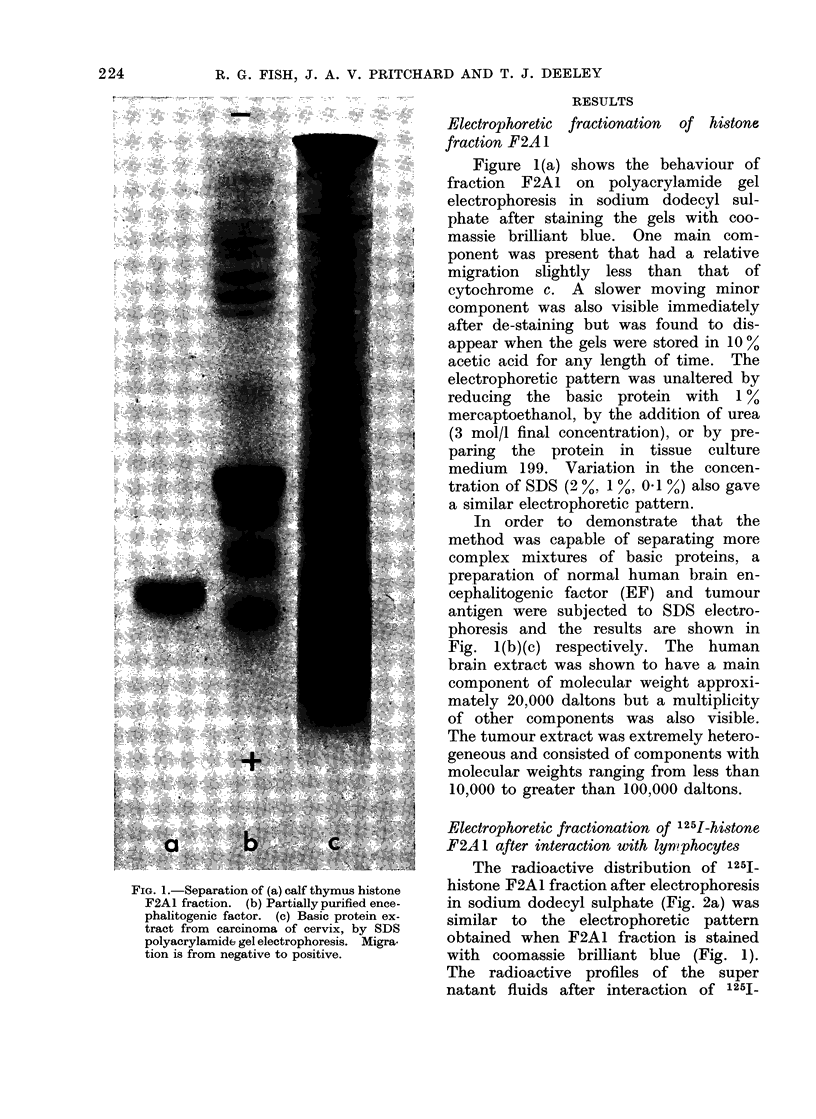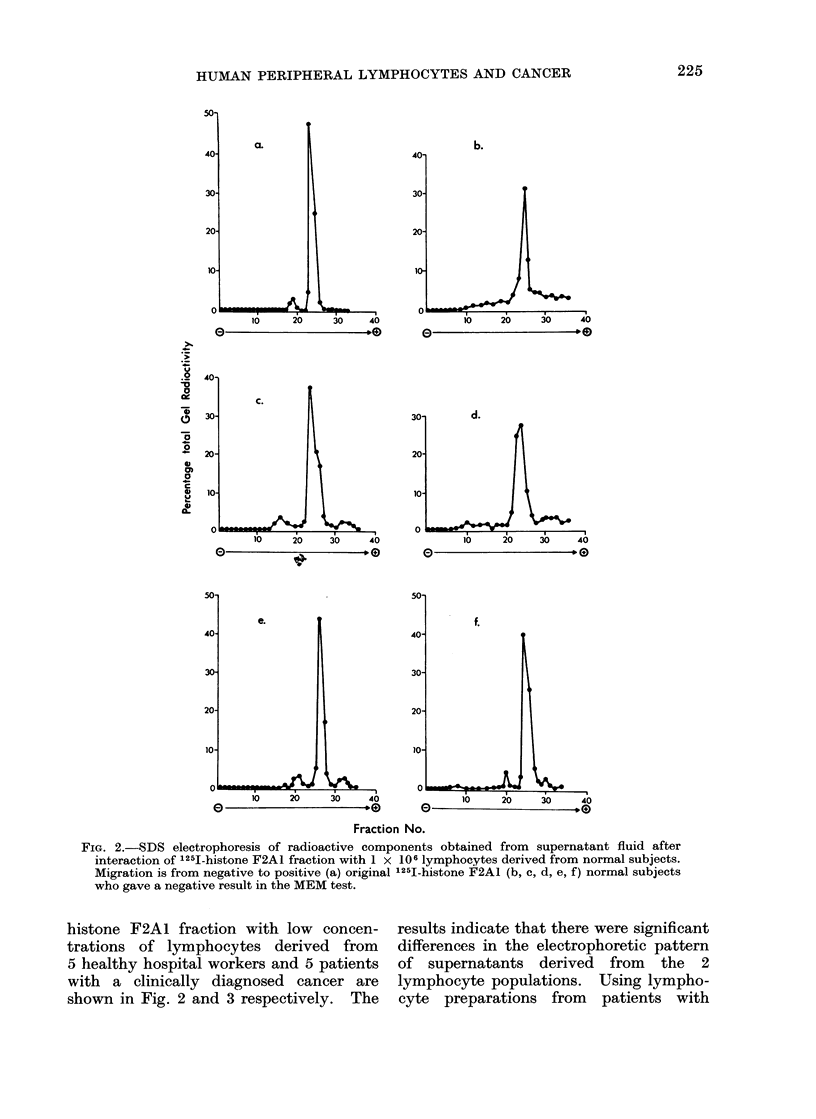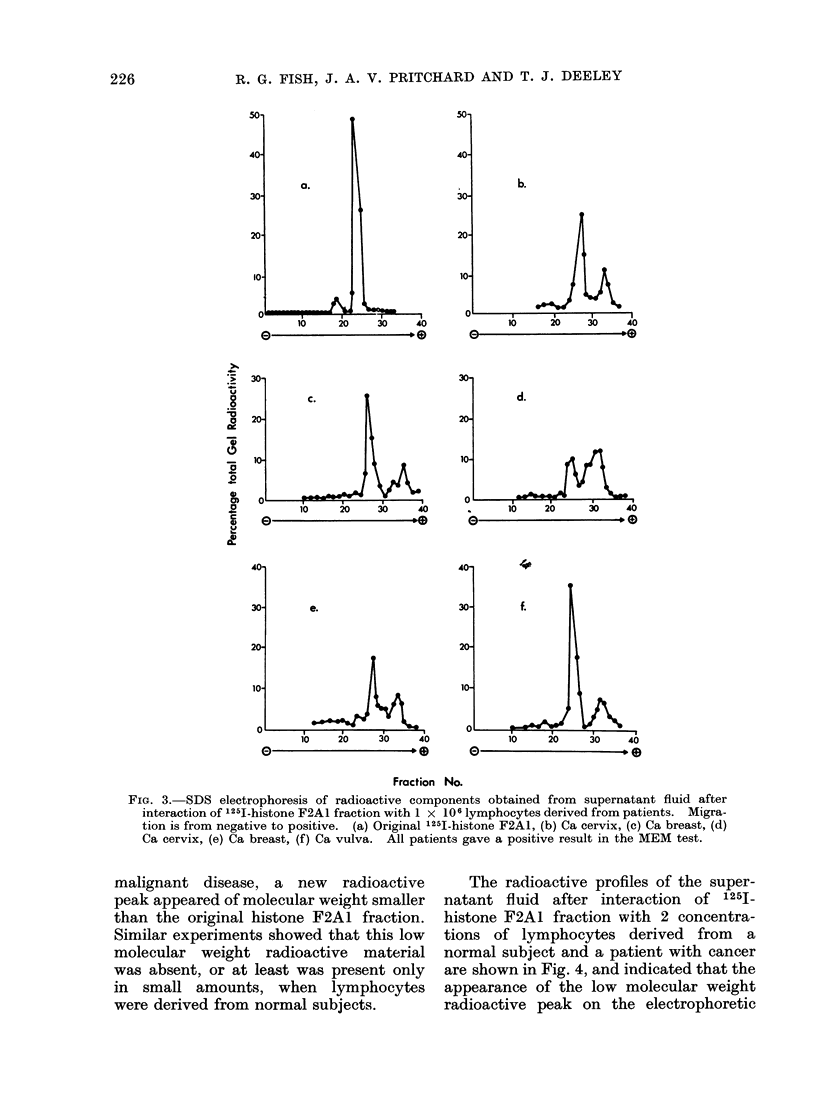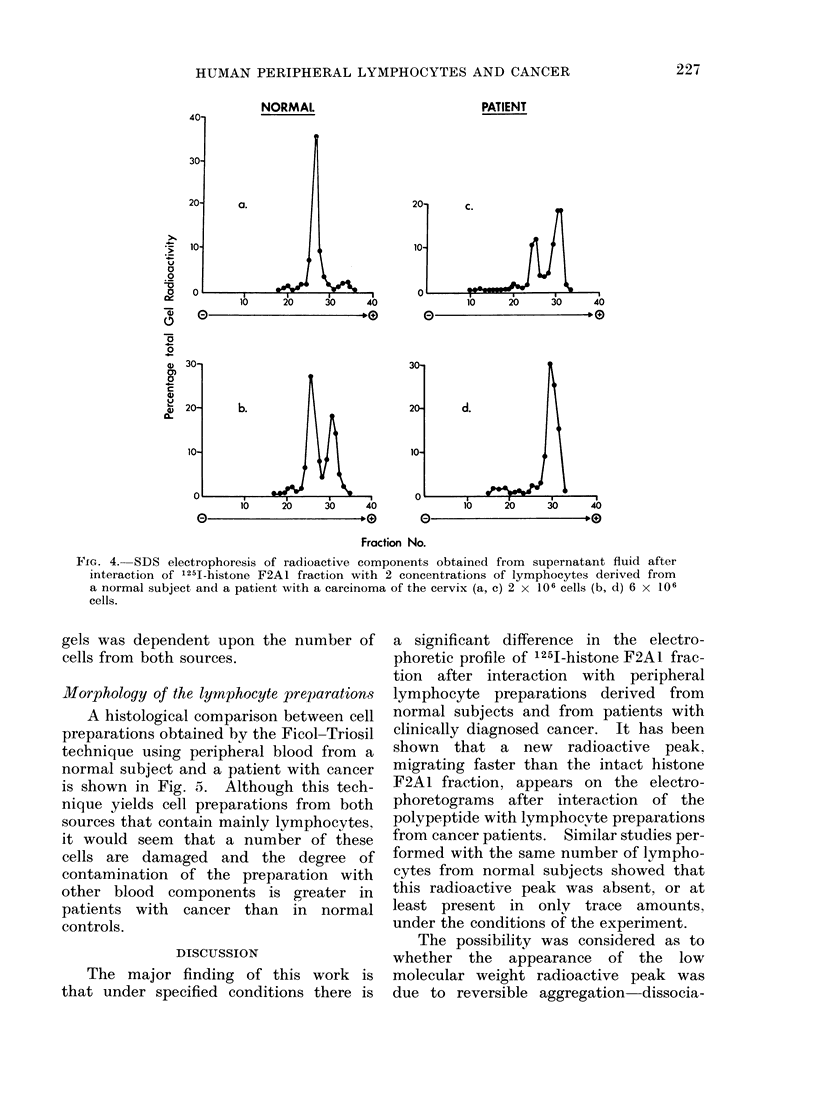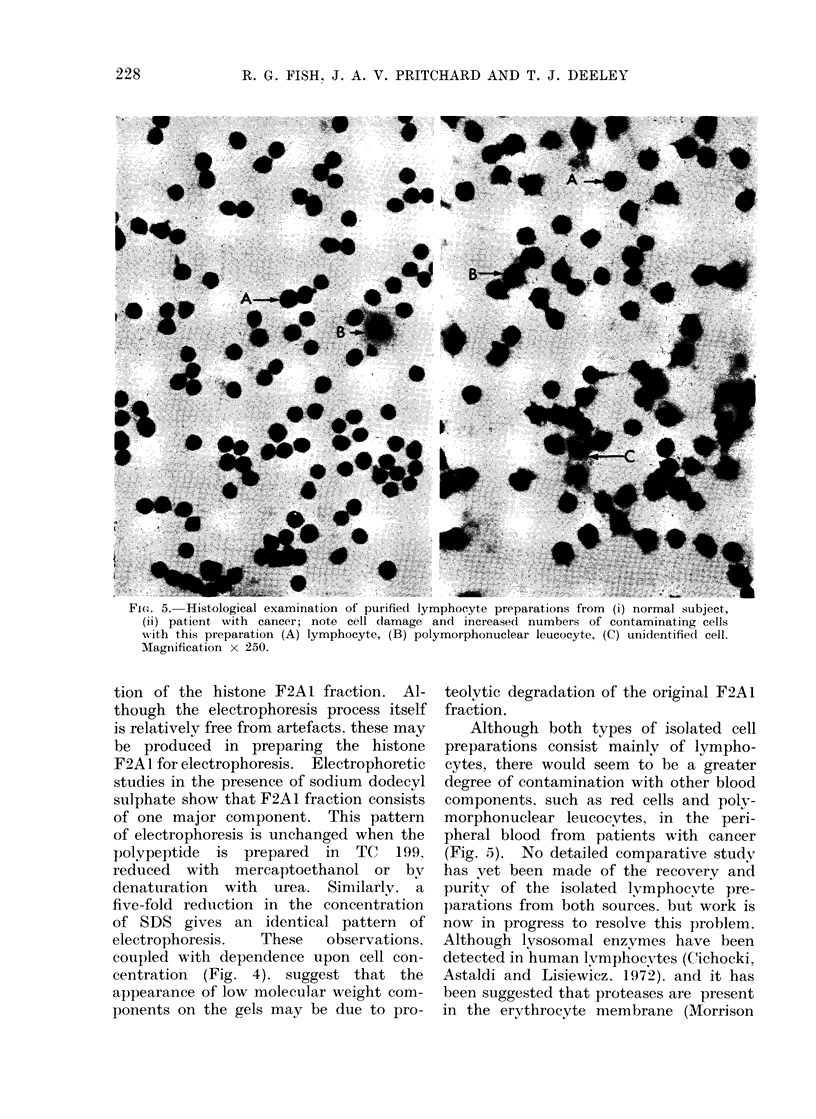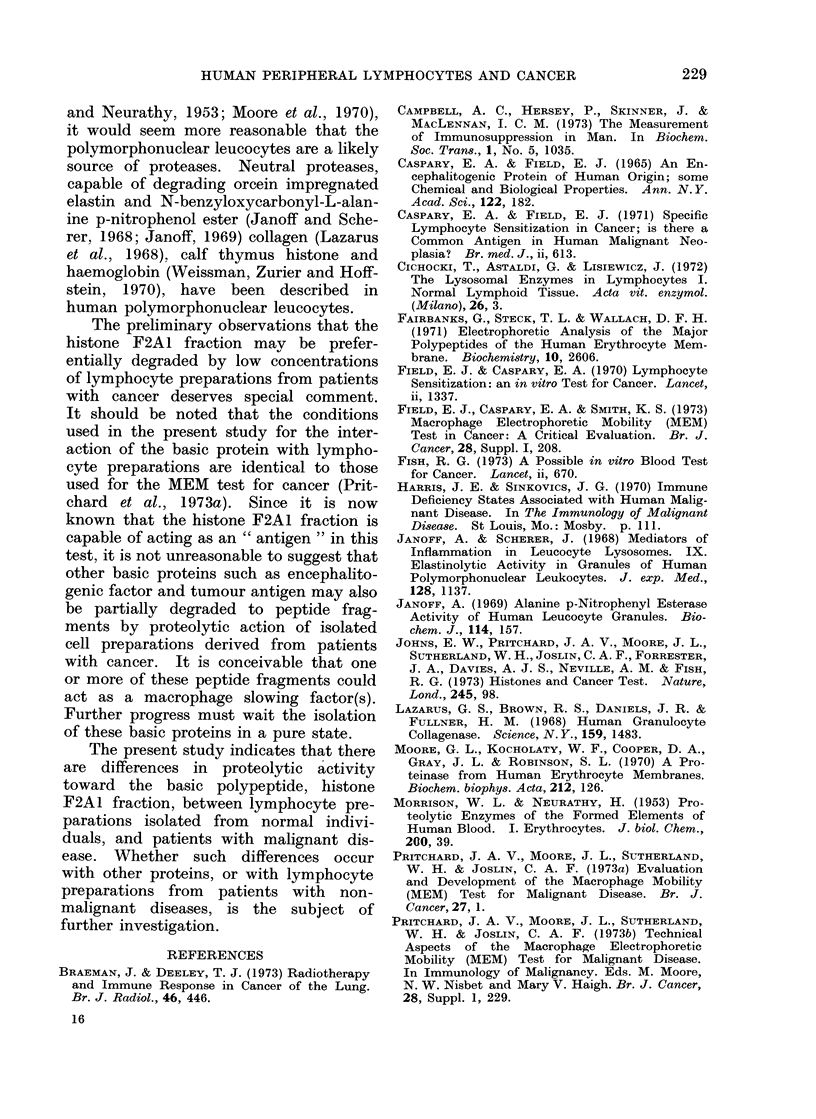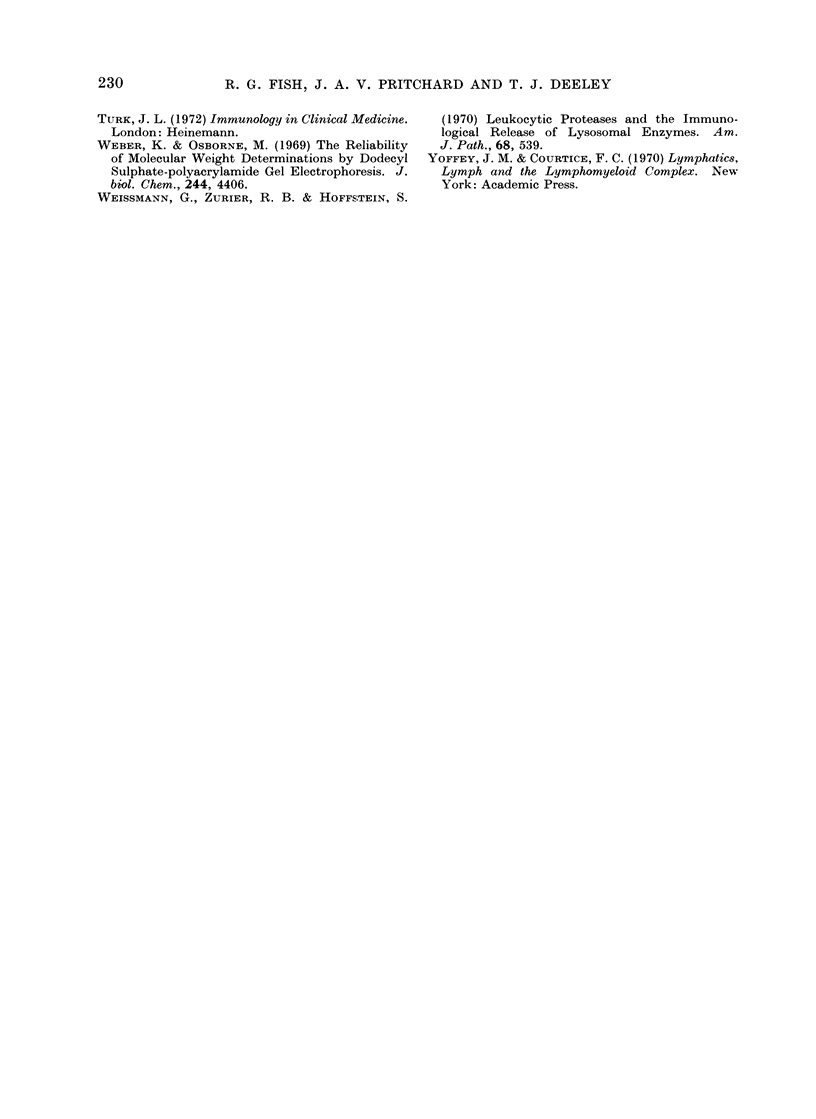# Human Peripheral Lymphocytes and Cancer

**Published:** 1974-09

**Authors:** R. G. Fish, J. A. V. Pritchard, T. J. Deeley

## Abstract

**Images:**


Br. J. Cancer (1974) 30, 222

### HUMAN PERIPHERAL LYMPHOCYTES AND CANCER

### IN VITRO STUDIES ON THE BASIC PROTEIN, HISTONE F2A1 FRACTION

R. G. FISH, J. A. V. PRITCHARD AND T. J. DEELEY

*From the Tenovus Laboratories, Velindre Hospital, Whitchurch, Cardiff*

Received 29 April 1974. Accepted 29 May 1974

**Summary.—The interaction of the highly purified basic protein " antigen ", calf thymus histone F2A1 fraction, with peripheral lymphocytes isolated from patients with cancer and from normal subjects has been studied. Analysis, by SDS polyacrylamide gel electrophoresis of the basic protein remaining in the supernatant fluid after interaction with low concentrations of lymphocytes from patients showed the presence of a component(s) of molecular weight smaller than the original histone F2A1 fraction. Similar experiments using lymphocytes derived from normal subjects indicated that this component(s) is absent, or at least is present in only small amounts. This difference could be partially abolished by using high concentrations of cell preparations. It is suggested that the observed difference is due at least in part, to differences in protease activity between the two preparations. The possible significance of these findings in relation to the macrophage electrophoretic mobility test for cancer is discussed.**

### INTRODUCTION

IN RECENT YEARS much interest has been focused on the interaction of a variety of antigenic materials with peripheral lymphocytes isolated from patients with cancer. For instance, the *in vitro* interaction of lymphocytes with glycoproteins from plant tissue (Yoffey and Courtice, 1970) and extracts from microorganisms (Turk, 1972) has been utilized to assess the immunological status of patients with neoplasias of the reticuloendothelial and lymphoid system (Harris and Sinkovics, 1970) and the immune response of patients with a variety of malignancies during treatment with anticancer drugs (Campbell *et al.*, 1973) and radiotherapy (Braeman and Deeley, 1973). More recently, the *in vitro* interaction of several structurally related basic proteins with lymphocytes isolated from patients with neoplasia have been studied (Field and Caspary, 1970; Pritchard *et al.*, 1973*a*) and form the basis of an immunodiagnostic test, the macrophage electrophoretic mobility (MEM) test for all forms of cancer (Field, Caspary and Smith, 1973).

As part of a general plan to compare the *in vitro* interaction of protein antigens with lymphocyte preparations isolated from normal subjects and patients with malignant disease, the present study deals with some aspects of the interaction of calf thymus histone F2A1 fraction. This basic protein was chosen for two reasons. Firstly, it can be prepared in highly purified form. Secondly, it has recently been shown that the polypeptide has the ability to act as an " antigen " in the MEM test (Johns *et al.*, 1973). A preliminary report of part of the present work has been published (Fish, 1973).

### MATERIALS AND METHODS

Ficol was obtained from Pharmacia Fine Chemical Ltd, Sweden, and Triosil from Vestric Ltd, Runcorn, Cheshire. Tissue culture medium (TC 199) was a product of

Gibco, Grant Island Biological Company, California. Jenner's and Giemsa's stains, acrylamide, N,N,$N^1$,$N^1$ tetramethylethylenediamine, ammonium persulphate, N,$N^1$-methylenebisacrylamide, mercaptoethanol, urea, bromophenol blue, $NaH_2PO_42H_2O$ and anhydrous $Na_2HPO_4$ were obtained from B.D.H. Chemicals Ltd, Poole. Sodium dodecyl sulphate (SDS), specially pure from B.D.H. was recrystallized once from hot water. Coomassie blue was a product of Imperial Chemical Industries Ltd, England.

The proteins used as molecular weight markers for SDS gel calibration were cytochrome *c* (horse heart) and $\gamma$-globulin (bovine) from Koch-Light Ltd, England; pepsin (hog stomach) from Sigma Chemicals Co. Ltd, England; albumin (bovine plasma) from Armour Pharmaceuticals Co. Ltd, England; trypsin (beef pancreas) and myoglobin (horse heart) from B.D.H.

The basic protein used in the present study was an arginine rich polypeptide fraction F2A1 isolated from calf thymus histone. Unlabelled and $^{125}$I-labelled polypeptide (approximately 100 $\mu$Ci/mg) were prepared by, and obtained as a generous gift from Dr E. W. Johns, Chester Beatty Institute, London. Encephalitogenic factor (EF) and tumour antigen (basic protein extract derived from a patient with carcinoma of the cervix) were prepared by the method of Caspary and Field (1965, 1971).

*Preparation of lymphocytes.*—Venous blood (10–20 ml) was collected from patients with clinically diagnosed cancer who had not undergone any form of treatment, and from healthy hospital workers. The blood samples were defibrinated and lymphocytes were prepared by the Ficol–Triosil technique used for the MEM test for cancer (Pritchard *et al.*, 1973*b*). After washing the cell preparations 3 times with TC 199, the pellets were resuspended in the same media. Aliquots of the cell suspensions were taken for histological examination and lymphocyte counting, using the Jenner–Giemsa staining procedure.

*Lymphocyte incubation studies.*—A sample of an appropriate diluted cell suspension (500 $\mu$l) in TC 199 was incubated with 60 $\mu$g of radioactively labelled F2A1 fraction for 90 min at room temperature. The suspension was then centrifuged at 1500 *g* for 10 min using an M.S.E. bench centrifuge with a swinging bucket rotor; the supernatant fluid was carefully removed and the pellet discarded.

*Preparation of samples for SDS electrophoresis.*—The protein samples and supernatant fluids were incubated at 37°C for 2 h in 10 mmol/l sodium phosphate buffer, pH 7·0 containing SDS at a final concentration of 0·5%. The samples (50 $\mu$l) were then added to 100 $\mu$l aliquots of a tracking dye mixture (50 $\mu$l 0·05% bromophenol blue, 500 $\mu$l 10 mmol/l sodium phosphate buffer pH 7·0 containing 0·5% SDS and 10 drops of glycerol); 50 $\mu$l or less of this protein mixture was applied to polyacrylamide gels.

*Electrophoretic conditions.*—Electrophoresis was performed essentially by the method of Weber and Osborne (1969). Polyacrylamide gels (5 × 75 mm) of 10% acrylamide solution in sodium phosphate buffer (8·8 g $NaH_2PO_4$; 20·5 g $Na_2HPO_4$ per litre, pH 6·9) containing 0·2% SDS, were used for all experiments. An analytical disc gel electrophoresis apparatus with a constant current power supply (Shandon Scientific Co. Ltd, London) was used for all fractionations. Reservoir buffers consisted of gel buffer diluted 1 : 1 with water. Electrophoresis was performed for $2\frac{1}{2}$–3 h at a constant current of 8 mA per gel with the positive electrode in the lower chamber.

*Staining and de-staining of gels.*—After electrophoresis, the gels were removed from the glass tubes and the total gel length and the migration distance of the tracking dye noted. The gels were then stained for protein with coomassie brilliant blue by the method described by Fairbanks, Steck and Wallach (1971). The relative migration of the protein standards and the histone fraction were calculated relative to the tracking dye.

*Measurement of gel radioactivity.*—After electrophoresis, gels containing $^{125}$I components were immediately cut laterally into segments by means of a razor blade and a metal stop with a 2 mm spacer. This simple procedure allowed reasonably uniform sections of approximately 2 mm width to be obtained. The segments were placed in plastic vials and the radioactivity in the fractions measured twice for 100 s in a well gamma counter. In some experiments radioactivity was monitored twice for 1000 s in an automatic gamma counter.

FIG. 1.—Separation of (a) calf thymus histone F2A1 fraction. (b) Partially purified encephalitogenic factor. (c) Basic protein extract from carcinoma of cervix, by SDS polyacrylamide gel electrophoresis. Migration is from negative to positive.

### RESULTS

*Electrophoretic fractionation of histone fraction F2A1*

Figure 1(a) shows the behaviour of fraction F2A1 on polyacrylamide gel electrophoresis in sodium dodecyl sulphate after staining the gels with coomassie brilliant blue. One main component was present that had a relative migration slightly less than that of cytochrome *c*. A slower moving minor component was also visible immediately after de-staining but was found to disappear when the gels were stored in 10% acetic acid for any length of time. The electrophoretic pattern was unaltered by reducing the basic protein with 1% mercaptoethanol, by the addition of urea (3 mol/l final concentration), or by preparing the protein in tissue culture medium 199. Variation in the concentration of SDS (2%, 1%, 0·1%) also gave a similar electrophoretic pattern.

In order to demonstrate that the method was capable of separating more complex mixtures of basic proteins, a preparation of normal human brain encephalitogenic factor (EF) and tumour antigen were subjected to SDS electrophoresis and the results are shown in Fig. 1(b)(c) respectively. The human brain extract was shown to have a main component of molecular weight approximately 20,000 daltons but a multiplicity of other components was also visible. The tumour extract was extremely heterogeneous and consisted of components with molecular weights ranging from less than 10,000 to greater than 100,000 daltons.

*Electrophoretic fractionation of $^{125}I$-histone F2A1 after interaction with lymphocytes*

The radioactive distribution of $^{125}$I-histone F2A1 fraction after electrophoresis in sodium dodecyl sulphate (Fig. 2a) was similar to the electrophoretic pattern obtained when F2A1 fraction is stained with coomassie brilliant blue (Fig. 1). The radioactive profiles of the super natant fluids after interaction of $^{125}$I-

FIG. 2.—SDS electrophoresis of radioactive components obtained from supernatant fluid after interaction of $^{125}$I-histone F2A1 fraction with $1 \times 10^6$ lymphocytes derived from normal subjects. Migration is from negative to positive (a) original $^{125}$I-histone F2A1 (b, c, d, e, f) normal subjects who gave a negative result in the MEM test.

histone F2A1 fraction with low concentrations of lymphocytes derived from 5 healthy hospital workers and 5 patients with a clinically diagnosed cancer are shown in Fig. 2 and 3 respectively. The results indicate that there were significant differences in the electrophoretic pattern of supernatants derived from the 2 lymphocyte populations. Using lymphocyte preparations from patients with

FIG. 3.—SDS electrophoresis of radioactive components obtained from supernatant fluid after interaction of $^{125}$I-histone F2A1 fraction with 1 $\times$ $10^6$ lymphocytes derived from patients. Migration is from negative to positive. (a) Original $^{125}$I-histone F2A1, (b) Ca cervix, (c) Ca breast, (d) Ca cervix, (e) Ca breast, (f) Ca vulva. All patients gave a positive result in the MEM test.

malignant disease, a new radioactive peak appeared of molecular weight smaller than the original histone F2A1 fraction. Similar experiments showed that this low molecular weight radioactive material was absent, or at least was present only in small amounts, when lymphocytes were derived from normal subjects.

The radioactive profiles of the supernatant fluid after interaction of $^{125}$I-histone F2A1 fraction with 2 concentrations of lymphocytes derived from a normal subject and a patient with cancer are shown in Fig. 4, and indicated that the appearance of the low molecular weight radioactive peak on the electrophoretic

FIG. 4.—SDS electrophoresis of radioactive components obtained from supernatant fluid after interaction of $^{125}$I-histone F2A1 fraction with 2 concentrations of lymphocytes derived from a normal subject and a patient with a carcinoma of the cervix (a, c) 2 × $10^6$ cells (b, d) 6 × $10^6$ cells.

gels was dependent upon the number of cells from both sources.

*Morphology of the lymphocyte preparations*

A histological comparison between cell preparations obtained by the Ficol–Triosil technique using peripheral blood from a normal subject and a patient with cancer is shown in Fig. 5. Although this technique yields cell preparations from both sources that contain mainly lymphocytes, it would seem that a number of these cells are damaged and the degree of contamination of the preparation with other blood components is greater in patients with cancer than in normal controls.

### DISCUSSION

The major finding of this work is that under specified conditions there is a significant difference in the electrophoretic profile of $^{125}$I-histone F2A1 fraction after interaction with peripheral lymphocyte preparations derived from normal subjects and from patients with clinically diagnosed cancer. It has been shown that a new radioactive peak, migrating faster than the intact histone F2A1 fraction, appears on the electrophoretograms after interaction of the polypeptide with lymphocyte preparations from cancer patients. Similar studies performed with the same number of lymphocytes from normal subjects showed that this radioactive peak was absent, or at least present in only trace amounts, under the conditions of the experiment.

The possibility was considered as to whether the appearance of the low molecular weight radioactive peak was due to reversible aggregation—dissocia-

FIG. 5.—Histological examination of purified lymphocyte preparations from (i) normal subject, (ii) patient with cancer; note cell damage and increased numbers of contaminating cells with this preparation (A) lymphocyte, (B) polymorphonuclear leucocyte, (C) unidentified cell. Magnification × 250.

tion of the histone F2A1 fraction. Although the electrophoresis process itself is relatively free from artefacts, these may be produced in preparing the histone F2A1 for electrophoresis. Electrophoretic studies in the presence of sodium dodecyl sulphate show that F2A1 fraction consists of one major component. This pattern of electrophoresis is unchanged when the polypeptide is prepared in TC 199, reduced with mercaptoethanol or by denaturation with urea. Similarly, a five-fold reduction in the concentration of SDS gives an identical pattern of electrophoresis. These observations, coupled with dependence upon cell concentration (Fig. 4), suggest that the appearance of low molecular weight components on the gels may be due to proteolytic degradation of the original F2A1 fraction.

Although both types of isolated cell preparations consist mainly of lymphocytes, there would seem to be a greater degree of contamination with other blood components, such as red cells and polymorphonuclear leucocytes, in the peripheral blood from patients with cancer (Fig. 5). No detailed comparative study has yet been made of the recovery and purity of the isolated lymphocyte preparations from both sources, but work is now in progress to resolve this problem. Although lysosomal enzymes have been detected in human lymphocytes (Cichocki, Astaldi and Lisiewicz, 1972), and it has been suggested that proteases are present in the erythrocyte membrane (Morrison and Neurathy, 1953; Moore *et al.*, 1970), it would seem more reasonable that the polymorphonuclear leucocytes are a likely source of proteases. Neutral proteases, capable of degrading orcein impregnated elastin and N-benzyloxycarbonyl-L-alanine p-nitrophenol ester (Janoff and Scherer, 1968; Janoff, 1969) collagen (Lazarus *et al.*, 1968), calf thymus histone and haemoglobin (Weissman, Zurier and Hoffstein, 1970), have been described in human polymorphonuclear leucocytes.

The preliminary observations that the histone F2A1 fraction may be preferentially degraded by low concentrations of lymphocyte preparations from patients with cancer deserves special comment. It should be noted that the conditions used in the present study for the interaction of the basic protein with lymphocyte preparations are identical to those used for the MEM test for cancer (Pritchard *et al.*, 1973*a*). Since it is now known that the histone F2A1 fraction is capable of acting as an " antigen " in this test, it is not unreasonable to suggest that other basic proteins such as encephalitogenic factor and tumour antigen may also be partially degraded to peptide fragments by proteolytic action of isolated cell preparations derived from patients with cancer. It is conceivable that one or more of these peptide fragments could act as a macrophage slowing factor(s). Further progress must wait the isolation of these basic proteins in a pure state.

The present study indicates that there are differences in proteolytic activity toward the basic polypeptide, histone F2A1 fraction, between lymphocyte preparations isolated from normal individuals, and patients with malignant disease. Whether such differences occur with other proteins, or with lymphocyte preparations from patients with non-malignant diseases, is the subject of further investigation.

### REFERENCES

BRAEMAN, J. & DEELEY, T. J. (1973) Radiotherapy and Immune Response in Cancer of the Lung. *Br. J. Radiol.*, **46**, 446.

CAMPBELL, A. C., HERSEY, P., SKINNER, J. & MACLENNAN, I. C. M. (1973) The Measurement of Immunosuppression in Man. In *Biochem. Soc. Trans.*, **1**, No. 5, 1035.

CASPARY, E. A. & FIELD, E. J. (1965) An Encephalitogenic Protein of Human Origin; some Chemical and Biological Properties. *Ann. N.Y. Acad. Sci.*, **122**, 182.

CASPARY, E. A. & FIELD, E. J. (1971) Specific Lymphocyte Sensitization in Cancer; is there a Common Antigen in Human Malignant Neoplasia? *Br. med. J.*, ii, 613.

CICHOCKI, T., ASTALDI, G. & LISIEWICZ, J. (1972) The Lysosomal Enzymes in Lymphocytes I. Normal Lymphoid Tissue. *Acta vit. enzymol.* (*Milano*), **26**, 3.

FAIRBANKS, G., STECK, T. L. & WALLACH, D. F. H. (1971) Electrophoretic Analysis of the Major Polypeptides of the Human Erythrocyte Membrane. *Biochemistry*, **10**, 2606.

FIELD, E. J. & CASPARY, E. A. (1970) Lymphocyte Sensitization: an *in vitro* Test for Cancer. *Lancet*, ii, 1337.

FIELD, E. J., CASPARY, E. A. & SMITH, K. S. (1973) Macrophage Electrophoretic Mobility (MEM) Test in Cancer: A Critical Evaluation. *Br. J. Cancer*, **28**, Suppl. I, 208.

FISH, R. G. (1973) A Possible *in vitro* Blood Test for Cancer. *Lancet*, ii, 670.

HARRIS, J. E. & SINKOVICS, J. G. (1970) Immune Deficiency States Associated with Human Malignant Disease. In *The Immunology of Malignant Disease*. St Louis, Mo.: Mosby. p. 111.

JANOFF, A. & SCHERER, J. (1968) Mediators of Inflammation in Leucocyte Lysosomes. IX. Elastinolytic Activity in Granules of Human Polymorphonuclear Leukocytes. *J. exp. Med.*, **128**, 1137.

JANOFF, A. (1969) Alanine p-Nitrophenyl Esterase Activity of Human Leucocyte Granules. *Biochem. J.*, **114**, 157.

JOHNS, E. W., PRITCHARD, J. A. V., MOORE, J. L., SUTHERLAND, W. H., JOSLIN, C. A. F., FORRESTER, J. A., DAVIES, A. J. S., NEVILLE, A. M. & FISH, R. G. (1973) Histones and Cancer Test. *Nature, Lond.*, **245**, 98.

LAZARUS, G. S., BROWN, R. S., DANIELS, J. R. & FULLNER, H. M. (1968) Human Granulocyte Collagenase. *Science, N.Y.*, **159**, 1483.

MOORE, G. L., KOCHOLATY, W. F., COOPER, D. A., GRAY, J. L. & ROBINSON, S. L. (1970) A Proteinase from Human Erythrocyte Membranes. *Biochem. biophys. Acta*, **212**, 126.

MORRISON, W. L. & NEURATHY, H. (1953) Proteolytic Enzymes of the Formed Elements of Human Blood. I. Erythrocytes. *J. biol. Chem.*, **200**, 39.

PRITCHARD, J. A. V., MOORE, J. L., SUTHERLAND, W. H. & JOSLIN, C. A. F. (1973*a*) Evaluation and Development of the Macrophage Mobility (MEM) Test for Malignant Disease. *Br. J. Cancer*, **27**, 1.

PRITCHARD, J. A. V., MOORE, J. L., SUTHERLAND, W. H. & JOSLIN, C. A. F. (1973*b*) Technical Aspects of the Macrophage Electrophoretic Mobility (MEM) Test for Malignant Disease. In Immunology of Malignancy. Eds. M. Moore, N. W. Nisbet and Mary V. Haigh. *Br. J. Cancer*, **28**, Suppl. 1, 229.

TURK, J. L. (1972) *Immunology in Clinical Medicine.* London: Heinemann.

WEBER, K. & OSBORNE, M. (1969) The Reliability of Molecular Weight Determinations by Dodecyl Sulphate-polyacrylamide Gel Electrophoresis. *J. biol. Chem.*, **244**, 4406.

WEISSMANN, G., ZURIER, R. B. & HOFFSTEIN, S. (1970) Leukocytic Proteases and the Immunological Release of Lysosomal Enzymes. *Am. J. Path.*, **68**, 539.

YOFFEY, J. M. & COURTICE, F. C. (1970) *Lymphatics, Lymph and the Lymphomyeloid Complex.* New York: Academic Press.